# Evaluation of the impact of the voucher and accreditation approach on improving reproductive behaviors and RH status: Bangladesh

**DOI:** 10.1186/1471-2458-11-257

**Published:** 2011-04-22

**Authors:** Ubaidur Rob, Moshiur Rahman, Benjamin Bellows

**Affiliations:** 1 Population Council, House CES (B) 21, Road 118, Gulshan, Dhaka -1212, Bangladesh; 2 Population Council, General Accident House, Ralph Bunche Road, Nairobi, Kenya

## Abstract

**Background:**

Cost of delivering reproductive health services to low-income populations will always require total or partial subsidization by the government and/or development partners. Broadly termed "Demand-Side Financing" or "Output-Based Aid", includes a range of interventions that channel government or donor subsidies to the service user rather than the service provider. Initial findings from the few assessments of reproductive health voucher-and-accreditation programs suggest that, if implemented well, these programs have great potential for achieving the policy objectives of increasing access and use, reducing inequities and enhancing program efficiency and service quality. At this point in time, however, there is a paucity of evidence describing how the various voucher programs function in different settings, for various reproductive health services.

**Methods/Design:**

Population Council-Nairobi, funded by the Bill and Melinda Gates Foundation, intends to address the lack of evidence around the pros and cons of 'voucher and accreditation' approaches to improving the reproductive health of low income women in five developing countries. In Bangladesh, the activities will be conducted in 11 accredited health facilities where Demand Side Financing program is being implemented and compared with populations drawn from areas served by similar non-accredited facilities. Facility inventories, client exit interviews and service provider interviews will be used to collect comparable data across each facility for assessing readiness and quality of care. In-depth interviews with key stakeholders will be conducted to gain a deeper understanding about the program. A population-based survey will also be carried out in two types of locations: areas where vouchers are distributed and similar locations where vouchers are not distributed.

**Discussion:**

This is a quasi-experimental study which will investigate the impact of the voucher approach on improving maternal health behaviors and status and reducing inequities at the population level. We expect a significant increase in the utilization of maternal health care services by the accredited health facilities in the experimental areas compared to the control areas as a direct result of the interventions. If the voucher scheme in Bangladesh is found effective, it may help other countries to adopt this approach for improving utilization of maternity care services for reducing maternal mortality.

## Background

Stagnating indicators for several reproductive and child health conditions in many countries of Africa and Asia are a major concern for national governments and development partners striving to achieve the Millennium Development Goals (MDGs). Universally, these indicators, and especially maternal and infant morbidity and mortality, are poorest among low-income populations. Weak and inefficient health systems sustain these inequities in access to and use of essential health services. There remains an over-reliance on the public sector to deliver these services, supported by the beliefs that: i) these services are too costly for low-income individuals or families to purchase and so the state needs to subsidize the full cost of their supply, and ii) that the private sector is not willing or able to deliver services to low-income clients because of their inability to pay the full cost.

Recognizing that the cost of delivering reproductive health services to low-income populations will always require total or partial subsidization by the government and/or development partners, alternatives to the traditional 'supply-side' approach to financing service delivery are being explored. Broadly termed "demand-side-financing" (DSF) or "output-based aid" (OBA), these alternatives include a range of interventions that channel government or donor subsidies to the service user rather than the service provider [1,2]. The goal is to increase access to and use of key services by subsidizing the user with sufficient resources to enable them to purchase the required service and to choose a provider from a number of alternatives. Doing this stimulates a market for the services, which creates competition between providers thereby motivating improvements in access to and quality of services to be able to attract users empowered with the resources to pay. Providers that perform well by attracting and serving users are thus rewarded by receiving payments for their services that both fully cover the delivery costs and provide some additional funds as an economic incentive.

An OBA approach, therefore, uses explicit performance-based subsidies to motivate providers to deliver selected reproductive health services at a specified level of quality and at an affordable cost so that the economically disadvantaged are not excluded. Several interventions are currently being developed and tested, including franchising and contracting, social health insurance, conditional cash transfers and vouchers. Voucher programs are intended to achieve a number of policy goals: i) by reducing financial barriers, they can increase access to services generally, and reduce inequities by making them affordable to the poor and other underserved groups; ii) by accrediting several providers to offer the service at the same price, they can increase choice for clients; iii) by including more than one provider, competition for clients with vouchers can increase efficiency in delivery and possibly reduce prices further; and iv) by requiring a minimal level of quality to be accredited to redeem vouchers, quality of care can be improved.

Vouchers (or coupons) for reproductive health are not new; Taiwan and Korea successfully used them in the 1960s to increase access to family planning [3], and Nicaragua implemented two voucher projects for sexually transmitted infection (STI) services in 1995 [4]. Bhatia et al. [5] showed that voucher scheme could be an option for increasing the utilization of reproductive and child health services in India. In the Yunnan Province of China, a voucher scheme was introduced for low-income, pregnant women to enhance the utilization of maternal and child health services. This scheme covered the cost of antenatal care (ANC), delivery and postnatal care (PNC) as well as care of sick children. Findings show that voucher distribution has increased the utilization of treatment for childhood diarrhea among the poor [6]. In Mexico, families are encouraged to obtain preventive health care, participate in growth monitoring and nutrition supplements programs and attend health education programs to be eligible for cash transfer. The study findings suggest that the cash transfer component is associated with better outcomes in child health, growth and development [7]. Experience with a discount voucher for insecticide treated bed-nets in Tanzania showed higher utilization of bed-net by poor, pregnant women and young children [8,9]. More recently there has been a resurgence of interest in this type of OBA. Key development partners interested in voucher schemes for reproductive health include the World Bank, Canadian International Development Agency (CIDA), Department for International Development (DFID) and the German Government. Specifically, the German Development Bank (KfW) is currently supporting OBA programs for reproductive health in collaboration with government and non-government partners in Kenya and Uganda, and is planning to start similar programs in Tanzania, Bangladesh and Cambodia. Between them, these programs are or will pilot-test voucher schemes for deliveries assisted by skilled personnel, family planning, prevention and management of STIs, care for survivors of sexual assault and male circumcision services.

In brief, these programs generally establish a voucher management agency (VMA) that produces and distributes or sells subsidized vouchers to clients, who purchase a voucher for a specific service and usually at a price that has been determined to be affordable for the lowest-income clients. The program also identifies and invites a number of service providers (individually or within an organization, which can be public, non-profit or for-profit) to participate in the program. Those agreeing to participate can only do so if they can demonstrate being able to provide the service(s) at a specified standard of quality of care; once this is demonstrated, they are then accredited to participate subject to their accreditation being regularly reviewed. When the client needs the services, s/he then redeems the voucher for the specified service at one of the accredited providers/facilities. The service provider is then reimbursed the full cost of providing the service upon submission of the voucher and supporting evidence to the VMA; this reimbursement usually includes an additional amount of money that serves as a profit or incentive.

Initial findings from the few assessments of reproductive health Voucher and Accreditation (V&A) programs suggest that, if implemented well, they have great potential for achieving the policy objectives of increasing access and use, reducing inequities and enhancing program efficiency and service quality [10-12]. At this point in time, however, there is a paucity of evidence describing how the various V&A programs function in different settings, for various RH services and for services delivered through public, for-profit or non-profit organizations and how the V&A program affects the operational efficiency and business model used by service delivery organizations and individual providers. There is also limited understanding of their effect on the quality of care received by clients and on levels of service utilization, especially among the poor and underserved. And most importantly, there is no evidence to date on their impact on RH behaviors and status at the individual and population levels, especially on those health status indicators relevant for the MDGs.

### Bangladesh- Output Based Aid (OBA) Program

In 2004 the Ministry of Health and Family Welfare (MOHFW), Government of Bangladesh, with technical support from World Health Organization (WHO), developed a maternal health voucher scheme. The overall goal of the DSF Maternal Health Voucher pilot is to achieve MDG target for reducing maternal mortality. The purpose of the pilot is to increase utilization of quality maternal healthcare services particularly by poor women from designated providers in selected upazilas through demand side subsidies. The Directorate General of Health Services (DGHS) of the MOHFW, under the Health, Nutrition and Population Sector Program (HNPSP), embarked on piloting the DSF scheme in initially in 21 upazilas and expanded it to 33 upazilas by 2007 [13]. In the third phase, the Government of Bangladesh has planned to expand the activities to 11 additional upazilas (Table 1). The MOHFW acts as the VMA. Out of 21 upazilas, there is universal targeting in 9 upazilas, so that all pregnant women are able to participate in the voucher program; in 12 upazilas, there is means-testing under which only poor pregnant women were included. The MOHFW has the plan to expand it to 11 additional upazilas, which will be supported through pool fund. The implementation of the third phase will start from early 2010.

**Table 1:** DSF rollout

Phase	Start date	Coverage	Funding	End date
I	August 2006	2 upazilas	WHO+UNFPA for one year, followed by pool fund	June 2011 (HNPSP period)
I	March 2007	19 upazilas	Pool fund	June 2011
II	Nov-Dec 2007	12 upazilas	Pool fund	June 2011
III	Early 2010	11 upazilas	Pool fund	June 2011
Total		44 upazilas		

DSF intends to transfer purchasing power to the poor to choose services directly from the accredited providers while the providers are reimbursed for their services from a special fund (Standing, Peters, and Varghese 2003). The selected beneficiaries under the pilot maternal health DSF scheme in Bangladesh receive a package of essential maternal health care services, as well as treatment of pregnancy and delivery related complications. In addition, the beneficiaries receive cash incentive of Taka 2,000 (US$29) for availing of safe delivery either in the facility or at home in presence of skilled birth attendant. They are also entitled to receive transportation cost of Taka 500 ($7) from home to the Upazila Health Complex (UHC), and additional Taka 500 ($7) for out-going referral to the District Hospital. This amount is disbursed in the form of unconditional cash grants. Besides, conditional cash transfer in the form of a gift box of about Taka 500 ($7) is provided to the pregnant women for safe delivery by accredited providers. The DSF scheme also allocates top-up funds to facilities, which are proportionately divided among staff and a facility maintenance fund. Generally, 50 percent of the top-up funds are deposited in the "seed fund" from where associated expendable costs are incurred. Thus, the DSF for maternal health care in Bangladesh is a combination of supply-side incentive for providers and demand-side cash transfer for clients.

German Technical Cooperation (GTZ) was involved in the DSF scheme through the Health Economics Unit (HEU) of MOHFW. HEU is the focal point of the MOHFW to conduct policy oriented, evidence-based and expenditure tracking studies to help and review policy formulation. HEU with support from GTZ undertook a rapid assessment of the pilot project in order to review the implementation and progress of the voucher scheme. Findings revealed increased service utilization among the target group with little impact on service quality or improvement, as well as serious concerns over unconditional cash transfer to clients for safe delivery including misreporting of the safe delivery for financial gain, complex management arrangement, problems of delay in disbursement mechanism through bank and unofficial charges from the providers' incentive [13,14]. However, there is lack of baseline information in both the assessments since evaluation was undertaken after inclusion of the program. Population Council, funded by the Bill and Melinda Gates Foundation (BMGF), will address this lack of evidence and increase awareness on the pros and cons of the voucher and accreditation approach to improve the maternal health of low income women in Bangladesh in the selected 11 new DSF upazilas.

This protocol describes an evaluation of the impact and effectiveness of the V&A approach in Bangladesh. The study has two specific aims:

#### 1. Assess the effect of the V&A approach on increasing access to, quality of, and reducing inequities in the use of selected RH services

Population Council - Dhaka office will:

a) Undertake health facility assessments, including providers' technical competence, skills and time-utilization, and clients' perceptions of quality of care at specified intervals at V&A and non-V&A facilities.

b) Undertake and compare time series analyses of service statistics for maternal and other RH services at V&A and non-V&A facilities. The data analyst in Population Council Dhaka offices will undertake time series analyses to prepare reports of, among others, trends in:

• Mean monthly number of clients obtaining RH services, by type, at V&A and non-V&A providers

• Mean monthly number and proportion of clients

• Obtaining RH services, by type, at V&A and non-V&A facilities

• Proportion of all health services at V&A facilities provided using the V&A approach.

• Proportion of RH services provided by V&A and non-V&A facilities at upazila level, by public and non-public sector.

c) Conduct client-provider interactions (CPI) and client exit interviews to measure the quality of maternal health care services during and immediately following the consultation with service providers during ANC visits, pre-discharge from the maternity unit, and six weeks postpartum visit.

d) Undertake in-depth interviews with key informants to gain a deeper understanding into the motivations, perceptions, and priorities of the service providers regarding V&A.

e) Review and strengthen the management information systems (MIS) with the Bangladesh DSF project (implementers of Phase 2 and VMA) to ensure that data necessary for measuring indicators are collected and to monitor and support routine collection of service statistics by V&A and non-V&A providers. Population Council staff will participate in meetings between the program implementing organization and the relevant service organizations, to ensure that the data requirements for measuring the provision and use of RH services are incorporated in revisions of the existing MIS. The MIS is expected to generate facility-level data such as the facility cesarean section rates, the proportion of still births and case fatality rates during delivery, all of which will be compared with upazila and national indicators.

##### Broad indicators

• Knowledge: provider competence; patient recognition of signs and symptoms of illness

• Utilization: RH service utilization; client load; client socio-economic profile; and market share for V&A services

• Quality: RH service quality as measured by facility readiness; information provision; compliance with norms; follow-up support; client perceptions

• Costs: out-of-pocket expenses; and expenses on voucher services

• Disease burden: proportion of complicated pregnancies.

#### 2. Evaluate the impact of the V&A approach on improving RH behaviors and status and reducing the inequities at the population level

The second aim of this research is to conduct and compare population-level surveys among representative samples of women stratified by socio-economic status in geographic areas served by V&A and non-V&A facilities in the selected upazilas. The study will also compare concentration index scores for selected RH indicators calculated from the data collected between V&A and non-V&A populations. The concentration index is a widely used indicator for quantifying the degree of income related inequality in a specific health indicator and will be used to provide evidence of the extent to which V&A approach reduces inequities.

Population-level surveys provide the opportunity to measure reproductive health indicators, including both reported health conditions and behaviors, among populations being served by a V&A program and comparable populations not served by the V&A program. Statistical comparisons between these indicators can then be used to detect any differences between the health status indicators of the populations and determine whether differences were due to the V&A program.

The survey will measure a set of indicators describing respondents' socio-demographic characteristics, health-seeking behaviors by health condition, RH conditions and behaviors relevant to the service being evaluated, experiences and perceptions of RH services received, and economic variables (include willingness and ability to pay) that permit the concentration index to be calculated. RH indicators will include measures of pregnancy and birth-related complications, physical and laboratory examination during pregnancy and post-partum, intake of vitamin A capsule, intake of iron syrup/tablet, TT vaccination, antenatal, delivery and postnatal services, among others.

The study expected to have impact on

• Intervention-dependent RH outcomes (pregnancy- and birth-related complications, physical and laboratory examination during pregnancy and post-partum)

• RH-related care behaviors (ANC, skilled delivery, postnatal care, LAM, breastfeeding, intake of vitamin A capsule, intake of iron syrup/tablet, TT vaccination)

• Awareness of RH issues, use of services, and expectations for use of services, and

• Inequities in experience of these outcomes and use of RH services.

## Methods/Design

There are two types of surveys that will be undertaken using the same design: population surveys and facility assessments. A quasi-experimental design will be followed in which surveys are undertaken among the target population for the V&A program before and after its introduction and also among an equivalent comparison population living in areas not served by a V&A program in order to control for potential time dependent confounding effect.

### Facilities

Facilities will be the primary sampling unit to measure access to and quality of care and service statistics. The upazila level administrative unit will be used to generate clusters of V&A accredited facilities and non-V&A facilities. These two sets of facilities will be matched to maximize the likelihood of patients having similar social, cultural, economic characteristics, and RH behaviors and to ensure similar staff composition. A total of 11 upazila level health facilities will be accredited in the third phase for DSF scheme in Bangladesh. All of these facilities will be selected for experimental group and same number of matched control non-accredited facilities will be selected from same or nearby districts.

Given that the V&A facilities will be self-selected to the experimental group through choosing to participate in the V&A program, there is a strong likelihood that they will be different from those not choosing or not invited to participate. To maximize the equivalence of these groups, thereby enhancing the validity of the design, a sampling design known as pair-wise matching will be used. In this design, the V&A and non-V&A facilities will be matched on selected characteristics including number of available service providers, availability of logistics and equipment, medicines, support staff, number of wards and beds, presence of anesthetist and gynecologist, and availability of comprehensive emergency obstetric care services or basic obstetric care services. A composite score of the different characteristics will be developed - such as a propensity score to facilitate matching of V&A and non-V&A facilities. This will help minimize group differences across many characteristics.

### Service providers

The knowledge and experiences of service providers in both V&A and non-V&A facilities will be assessed. All eligible service providers available on the day of data collection at the facility will be interviewed to measure indicators concerning provision of maternal and neo-natal health (MNH) and RH services. Service providers responsible for maternal health/RH services within the facility will be eligible for inclusion.

At the time of the interview, the benefits and risks of their participation will be described to each provider and they will be assured that consenting to be interviewed is voluntary and all information provided will be kept in confidence. No-one, including their supervisor, will know what they say and their names will not appear on the questionnaire sheet. Although summary information and anonymous opinions from providers will later be presented, it will not be known who said what.

### Key informants

There are district, upazila and union level DSF committees to monitor the DSF scheme in Bangladesh. The study will also carry out in-depth interviews (IDIs) with these key informants. These will take place while teams conduct the facility-based assessments. These interviews will be used to gain a deeper understanding into the motivations, perceptions, and priorities of the service providers regarding V&A. The key informant IDIs will focus more specifically on:

• Services offered

• Attitudes towards V&A, including effects on workload

• Benefits and challenges of the V&A

• Perception of clients' views

• The referral system

• Other health care needs

• Reimbursement process

• Management issues

At the end of the IDIs, participants will be provided with any necessary information to complete their understanding of the nature of the study. The researcher will discuss with the participants their experience with the research in order to monitor any unforeseen negative effects or misconceptions.

### CPI surveys of quality of care for specific maternal health care consultations

Measurement of the quality of maternal health care services will be conducted at designated consultation times: during any ANC visit, pre-discharge from the maternity unit, and six weeks postpartum. Measurement will be done using cross-sectional surveys of observations of consultations and client exit interviews immediately following the consultation. Samples of clients attending each type of consultation will be recruited if they meet the following eligibility criteria:

• Are accessing maternity care including postnatal care, for themselves (and/or their babies), at delivery or one of the pre-discharge, one-week or six-week postpartum consultation times;

• Are aged over 18 years (the small proportion of clients that are less than 18 years will not justify the difficulties in obtaining parental/guardian permission for legal minors);

• Are aged below 45 years (the small proportion of women giving birth/accessing FP above this age will be excluded);

• Give their informed consent for their consultation to be observed and the key actions taken recorded, and to be interviewed on exiting from the consultation.

All women satisfying these inclusion criteria will be recruited until the required sample sizes have been reached.

### Client exit respondents

Client exit interviews will be conducted to measure the quality and satisfaction level of maternal health care services immediately following the consultation with service providers during ANC visits, pre-discharge from the maternity unit, and six weeks postpartum visit. Samples of clients attending each type of consultation will be recruited if they meet the following eligibility criteria:

• Are accessing essential maternity care including ANC, PNC, for themselves (and/or their babies), at pre-discharge following delivery and six-week postpartum consultation times;

• Are aged over 18 years;

• Are aged below 45 years;

• Provide consent for exit interview.

All women satisfying these inclusion criteria will be recruited until the required sample sizes have been reached. For both CPI and client exit interviews, any women wishing to withdraw at any point during the interview will be allowed to do so; they will have been informed that their withdrawal will not affect their right to obtain services. Using a structured consent form (administered in their preferred language), all women will be informed about the objectives, procedures, benefits, and risks of the study at the time of interview.

### Population survey respondents

Surveys will take place early 2010 and early 2012 to compare patterns of service use and perception and to compare any differences between communities that have ready access to the voucher and communities that do not have access. Each fieldworker has a catchment area and they visit households regularly for collecting the list of pregnant women using their register book. This register book will be used as sampling frame to draw required number of sample through simple random sampling procedure. Respondents will be interviewed if they meet the following eligibility criteria:

• Became pregnant or delivered in the last one year preceding the survey.

• Are aged over 18 years (the small proportion of clients that are less than 18 years will not justify the difficulties in obtaining parental/guardian permission for legal minors);

• Are aged below 45 years (the small proportion of women giving birth/accessing FP above this age will be excluded);

• Provide consent for interviews.

### Sample size

The national proportion of facility-based births is 14.6% of all births. The national figure has been considered as baseline level of facility-based births in the voucher region. To detect a 12% increase in the proportion of facility-based births, 1046 experimental subjects and 1046 control subjects are required to be able to reject the null hypothesis that the proportion of facility-based births for experimental and control subjects are equal with probability (power) 0.8. The Type I error probability associated with this test of this null hypothesis is 0.05. An uncorrected chi-squared statistic will be used to evaluate this null hypothesis.

### Selection process of survey respondents

Survey respondents will be drawn from the facility catchment areas in the selected experimental and control sites. Depending on the final number of facilities enrolled, a complete list of unions (lowest administrative unit) in the catchment areas will be made and at least three unions out of nine unions from each upazila will be selected through probability proportional to size (PPS) where size is the total population or total number of pregnant women. Finally two villages will be selected from each union through PPS. Total 132 villages from 66 unions will be selected from 22 upazilas for interviews. A final list of survey villages will be drawn in this way. From each selected village, required number of respondents will be selected from the list of pregnant mothers prepared by the respective fieldworkers. Within each village, it is estimated that there are approximately 20 deliveries in 12 month period.

### Evaluation

#### 1. Assess the effect of the V&A approach on increasing access to, quality of, and reducing inequities in the use of, selected RH services

##### Undertake health facility assessments

Population Council will conduct two health facility assessments (HFAs) of the quality of care provided in GOB, NGO and private-for-profit facilities (NGO and private facility assessment depends on inclusion of these types of facilities in the proposed DSF phase). An initial assessment will be undertaken in both V&A and non-V&A facilities to determine the comparability of the facilities and to provide baseline measures of the quality of care. To determine the sustainability of the quality of care provided an additional assessment will be undertaken 18-24 months later after the baseline assessment. In addition, data collected through routine monitoring of service statistics (see below) will provide further information about the sustainability of the service configurations in terms of client load, services mix, client characteristics etc.

#### Data collection

Data collection procedures for facility assessments are as follows:

##### Facility inventory

An inventory of available resources to learn about the facility infrastructure, staffing numbers and skills mix, services provided, staff training undertaken, availability of equipment, commodities, test kits, stationary (client cards and notes), medications required to provide the services within the intervention will be undertaken. The head of the facility will be approached to assign a nurse/midwife to facilitate the work of researcher. The nurse/midwife will guide the researcher around the facility to observe and record all relevant information on a checklist.

##### Collecting service statistics

Service statistics and HMIS form will be collected throughout the project period from all selected facilities. The study will also review service statistics (related to routine program data) regarding utilization of maternal health care services for a 12 month period prior to the assessment visit. The number of new and continuing clients coming to a clinic for maternal health care services as well as other health services will be recorded. Monthly trends in the numbers of new and continuing ANC and PNC clients as well as for other services will be obtained from facility records. In addition, DSF program implementation cost will be analyzed from the facility for cost analysis.

##### Interviews with service providers

Service providers' interviews will be conducted to determine their knowledge and skills for MNH and other related RH services, as well as understand the organizational setup and description of related activities. Interviews will also ascertain their perceptions of barriers and operational challenges that may influence V&A clients' acceptance of services and the provider's attitudes towards the accreditation process. All providers related to maternal health care services will be approached for interview in each upazila and union level health facilities. It is estimated that approximately 15 service providers (10 from UHC and 5 from Health and Family Welfare Centers) will be eligible to participate in the study. This will give around 165 providers for experimental and comparison groups each (15 providers × 11 facilities). Finally, a total of 330 providers will be interviewed both from experimental and control areas. The same number of interviews will be carried out in the post-intervention period to measure the changes due to program.

##### Observation of client-provider interactions (CPI)

The CPI encompasses both the **process **(how clients are treated and whether they actively participate) and the **content **(what they are told, technical competence, accuracy of information, provision of essential information) of a consultation. After obtaining informed consent from the client, a structured non-participatory observation of the client-provider interaction will be undertaken to determine the quality of ANC, delivery and PNC services provided. CPI will be conducted until 100 randomly selected, ANC, delivery and PNC clients in each facility has been observed. Finally, 2200 clients (100 clients × 22 facilities) will be observed through CPI. This includes all voucher and non-voucher accredited facilities.

It is likely that observing client provider interaction may influence the result obtained on quality of care in a positive direction. To reduce this risk, the research team will spend more than one day at each site, so that the presence of the research team becomes more familiar and the behavior of the providers becomes more normative.

To measure the magnitude of changes in the quality of services provided, composite summary scores will be developed for a series of key indicators by aggregating the mean scores of key items being assessed for each individual client-provider interaction being observed. This scoring system will categorize whether an accepted standard of quality has been met or not. For each study group, a mean score will be calculated for each indicator and then a composite summary score will be calculated which will help to make statistical comparisons between experimental and control groups over time. Examples of the types of individual items and key indicators that will be used are given in Table 2.

**Table 2:** Examples of groups of key actions/indicators to make composite scores of quality of care

Quality of:	Observed provider actions:
a. Client - provider rapport (0-7)	Client greeted warmly, Discussed medical conditions, Asked if client understood information, Encouraged client to ask questions, Used client's name, Help in decision-making, Consultation time > 15 minutes

b. ANC counseling	Birth planning, danger signs, physical and laboratory examinations, vitamin A capsule, iron tablet/syrup, TT vaccination, infant feeding, fertility intentions

c. PNC counseling on danger signs since childbirth (0-10)	Ask about: bleeding since birth, color/smell of vaginal discharge, condition of perineum/CS scar, fever, headache or blurred vision, swelling in face, hands or feet, signs of thrombophlebitis, tiredness or breathlessness, convulsions or fits, LAM, breastfeeding

The proportion of women receiving an acceptable quality of service will be calculated in addition to the mean scores. This is because the mean score may be high if a small proportion of clients receive excellent services. The methodology to calculate the proportion of women receiving an acceptable quality is similar to the Lot Quality Assurance Sampling (LQAS) approach that has been used in Kenya and elsewhere for assessing quality [15]. LQAS follows the principle that an entire group (lot) of services is deemed poor quality if a certain proportion within a small sample does not reach a minimum standard. LQAS applies cumulative probabilities calculated with a binomial formula to select small sample sizes and decision criteria for judging a group of providers. In consultation with the Ministry of Health and Family Welfare the minimum standard for quality maternal health care services will be reviewed and adapted by building on existing guidelines and protocols and previous studies.

##### Client exit interviews

Exit client interviews will be conducted with married women aged 18-45 years who receive maternal, newborn and child health care services in all intervention and control areas. From 22 areas, a total of 2200 (100 clients × 22 facilities) women will be interviewed. The exit client interviews will provide information about accessibility to services, the clients' attitude towards receiving services from the facility, and quality of services received from the facility with regard to privacy, confidentiality, non-judgmental attitude and respect. To determine the sustainability of the quality of care provided, exit interviews will be undertaken periodically to determine the extent to which the quality of care has changed.

##### In-depth interviews with key informants

Interviews will be conducted with the DSF committee members and district and upazila level program mangers to determine their knowledge regarding voucher and accreditation approach as well as understand the organizational setup and operational issues of voucher. Interviews will also ascertain their perceptions of barriers and operational challenges that may influence V&A clients' acceptance of services and the provider's attitudes towards the accreditation process. In each DSF upazila, there are district, upazila and union level DSF committees to oversee and monitor the whole process of voucher scheme. All members who will be present during the data collection period will be interviewed. Approximately 110 key informants (10 key informants × 11 facility area) will be interviewed from intervention areas.

#### 2. Evaluate the impact of the voucher and accreditation (V&A) approach on improving RH behaviors and RH status and reducing the inequities at the population level

*Population based survey: *Population Council will conduct population-based surveys with a randomly selected sample of women aged 18-45 years from the catchment communities of selected study facilities. The intention of the population-based surveys is to build a view of the women's utilization of services using vouchers and V&A facilities in the region. A picture of the use of services will be invaluable in understanding the access to and use of services available to the target population. Similar cross sectional survey will be carried out in the catchment area of non V&A facilities. A second pair of surveys will be executed in the same areas in early 2012.

Using a structured written consent form (administered in their preferred language), all women will be informed about the objectives, procedures, benefits, and risks of the study. Informed consent will be obtained separately for each interview according to the informed consent procedure described in the appendices. Women will be asked standardized and sex-specific questions on access and use of services, attitudes, experiences and reasons for service use/non-use of V&A and RH issues. Population based study will provide support to measure changes or differences in characteristics, behavior and attitudes of voucher holders *vs. *non-voucher holders, as well as offer insights into preferences for the accredited services and reasons for use/non-use of these. Some of the questions may have the potential to cause distress to the women. Therefore, the research assistants will be carefully selected and will work under the supervision and support from the study coordinator. This will also provide an opportunity to validate several findings with the findings of exit client and CPI. Provisions will be made to train researchers to ensure that guidance on ethical conduct is clearly understood and implemented. Training of research assistants is likely to take a minimum of eight days including a pretest in the field. Table 3 describes examples of operational results and indicators to compare V&A and non-V&A health facilities and communities.

**Table 3:** Examples of operational results and indicators to be used to compare results from the V&A and non V&A health facilities and communities

Objective 1: To evaluate the impact of the V&A approach on improving reproductive health behaviors and status and reducing inequities at the population level		
**Results**	**Indicators**	**Data source**

Increase in clients using maternal health care services	Clients received ANC services from public health facility Clients received institutional safe delivery care (normal/vacuum/forceps/caesarean section) Clients received pregnancy related complications management from the designated facility Clients utilized PNC services Clients received facility-based care for managing life threatening complications Clients received key physical and laboratory examinations (weight, height, blood test, urine test, abdomen exam, sonogram or ultrasound, and anemia exam).	Service statistics Population survey

Improved attitudes of service providers towards poor women	Providers indicating non discriminatory attitudes Clients recommending services to others	Exit client interview CPI Population survey

Improved quality of services	Service waiting hours Round-the-clock service availability Maintaining privacy and confidentiality Availability of necessary service equipments and logistics	Client Provider Interaction Client exit Facility inventory

Reduced out-of-pocket expenses	Medicine cost Transport cost Unofficial charge by the providers	Population survey

Reduced disease burden	Proportion of untreated complicated pregnancies	Population survey

**Objective 2: To assess the effect of the V&A approach on increasing access to, quality of, and reducing inequities in the use of selected RH services**		

**Results**	**Indicators**	**Data source**

Increased knowledge and skills of service providers on maternal health care issues	Recite proper schedule of TT and child immunization Life-threatening complications management Referral conditions for life-threatening conditions	Interviews with service providers

Increased awareness of clients on maternal health care issues among all clients and poor clients	Complications during pregnancy, delivery and post-partum period Number of ANC visits and schedule Schedule of Vitamin A capsule and iron tablet or syrup Schedule of TT and child immunization Breastfeeding	Population survey

Increased utilization of maternal health care services	Clients received ANC services from public health facility Clients received institutional safe delivery care (normal/vacuum/forceps/caesarean section) Clients received pregnancy related complications management from the designated facility Clients utilized PNC services Clients received facility-based care for managing life threatening complications Clients received key physical and laboratory examinations (weight, height, blood test, urine test, abdomen exam, sonogram or ultrasound, and anemia exam).	Population survey

Improved patient satisfaction with health care experiences	Perceived barriers to accessing services: costs, distance, quality, waiting times, privacy, confidentiality, respect, stigma surrounding service	Population survey Client exit

Paper questionnaires or if possible net book will be used to capture quantitative data. Data from paper questionnaires will be keyed into Epidata 3.1/Access and exported into Stata 10/SPSS 14 for Windows for analysis. Data from net book will downloaded into an MS Access database before being exported into Stata 10/SPSS 14 for Windows for analysis. Tests of correlations between control and experimental or pre-intervention and post-intervention periods will be made at 1% and 5% level of significance. Qualitative data will be captured on paper and audio tapes and later typed into MS Word before conversion into text files for formatting and exporting into QSR N6/ATLAS.ti for analysis. Data stored on computers and associated hardware will be password-protected. Hard copies of questionnaires, anonymised transcriptions and tapes of the in-depth interviews will be stored securely in a locked cabinet, in accordance with the Population Council and country-specific data protection criteria.

## Discussion

This is a quasi-experimental study which will investigate the impact of the voucher approach on improving maternal health behaviors and status and reducing inequities at the population level. We expect a significant increase in the utilization of maternal health care services by the accredited health facilities in the experimental areas compared to the control areas as a direct result of the interventions. If the voucher scheme in Bangladesh is found effective, it may help other countries to adopt this approach for improving utilization of maternity care services for reducing maternal mortality.

### Ethical consideration and informed consent

#### Risks

There are a few risks involved with conducting this study. During the interviews, women will be asked a number of potentially sensitive questions, including potentially their reproductive behavior and perceptions of contraceptive use. Therefore, careful steps will be taken in the questionnaire design to minimize potential discomfort to our informants. The study tools will also be pre-tested among a small group of women with similar characteristics as the study population to identify potentially negative consequences and modified accordingly. To avoid the risk of others overhearing this information, interviews will be conducted in strictly private settings, and ample time will be allowed for data collection to ensure that privacy and confidentiality can be guaranteed.

Provisions will be made to train researchers to ensure that guidance on ethical conduct is clearly understood and implemented. Such training will include focused sessions and exercises regarding the meaning and process of informed consent, the importance of protecting the privacy of subjects, and confidentiality of the information obtained from them. The research team will also be trained to listen and observe intently without displaying any judgmental attitude towards information they receive from the informants and on other critical ethical issues on gathering information from the women.

Interviews will only be recorded after obtaining written informed consent from the interviewee. From the outset, it will be made clear to participants that they have a right to withdraw from the research at any time. All personal data will be treated confidentially. Data stored on computers and associated hardware will be password-protected. Hard copies of questionnaires will be stored securely in a locked cabinet for no longer than five years after the study has ended, in accordance with the Council Policy of Dhaka Office. At the end of the interview, participants will be provided, with any necessary information to complete their understanding of the nature of the research. The researcher will discuss with the participants their experience of the research in order to monitor any unforeseen negative effects or misconceptions.

#### Benefit

No immediate tangible benefit is likely to accrue to the subjects through their participation and this will be made clear when obtaining informed consent. However, the potential benefits to health care services and the women who use them of conducting this study will be described to the potential participants, so that they are fully aware that the data gathered will be used to provide recommendations to the MOHFW, as well as to the service providers and communities in other countries.

#### Confidentiality

Given the sensitive nature of the information to be gathered, protecting and respecting the confidentiality and privacy of our informants will be a critical consideration throughout the study. We intend to use individual in-depth interviewing in order to provide each informant with a greater assurance of confidentiality. The research teams will discuss and develop methods and procedural measures in relation to matters such as data recording style, personal identifiers, transcription and processing procedures, lifespan of unprocessed data, type and places of storage, and data safety and right of access. This will be developed prior to the onset of the research study. All data will be kept separately from identifying information and will be stored under locked files. Access to data will be strictly limited to the research team. All interviews will be conducted in private. Identifying details will not be included in the results presentation. Confidentiality will be maintained at all times through training interviewers, the meaning of confidentiality and ways of maintaining confidentiality during data collection and afterwards.

All data collection instruments will be kept under lock and key at all times in the field and in the Population Council's Dhaka office. All data collection instruments will contain a study ID number. Names will only be recorded for women agreeing to any follow-up visits to facilitate contact. This information, together with their contact information, will be recorded on a separate sheet and kept physically separate from the data collection instruments containing their information, and will only be linked to questionnaires by study ID numbers. Only the research team coordinator will have access to both the names and instruments.

Data collectors will be asked to work where they are not known personally, to respect privacy and ensure respondents' anonymity. Results of client, service provider interviews and key stakeholders will be presented in reports in an aggregated manner such that responses cannot be traced back to individuals. Population Council staff will visit the data collection site to ensure interviewers' adherence with confidentiality procedures.

As part of Population Council's monitoring program, the subjects participating in the research will be asked to acknowledge the possibility that an interview may be requested by a representative of Population Council to determine if informed consent occurred. Following a request for an interview, the subject will have the option of accepting or declining the interview.

#### Compensation

During follow-up interviews, a woman may not want others to know that she is participating in the study, and so we will offer all women the opportunity to hold the interview in a location of her choosing, and will reimburse travel costs to ensure that this is a viable option. The majority of clients will be interviewed at the clinic or during household surveys. However if/where our informants are requested to travel specifically for interview, the study will compensate informants (at an average rate of US $1.5 per study participant) for this inconvenience by providing transport refund to attend pre-arranged interview meetings. Similarly, service providers and key stakeholders will not receive any money except for the transportation cost to attend the meeting outside their point of service delivery.

#### Informed Consent

Structured verbal consent forms will be used in one-to-one interviews and the respondents will be informed about the study objectives, time needed for the interview, and risks and benefits. Potential participants will be assured that they have the right to withdraw from the interview at any point in time. They will also be informed that they have the right to refuse to answer any or all of the questions, even after consenting to participate in the study. All respondents will be informed that as part of the Population Council's policy on adherence to ethical procedures, a representative of the Population Council may request a private interview to confirm that the participant was properly informed about the study and procedure before giving consent. After being fully informed about the study objectives, procedures, benefit and risks, the respondents will be asked to give verbal consent if they wish to participate in the study. A trained interviewer will attest to their verbal informed consent to participate. Written informed consent will be obtained from the service providers and key stakeholders before conducting interviews following the standard Council procedures.

### Ethical approval

This project deals with some issues that are considered sensitive in nature (RH behavior). Ethical considerations in this project relate to risk and risk management, protecting confidentiality and anonymity of the informants, compensation, and obtaining consent and assent. The study protocol and instruments received approval by the Institutional Review Board of the Population Council. As prescribed by New York State and Federal laws, the Population Council requires that all studies, unless exempt, involving human subjects need to be reviewed by its Institutional Review Board (IRB) before the research is initiated. The members of the Institutional Review Board of the Population Council are: John Bongaarts, Chairman, Art Allen, Machelle Allen, Vanessa Cullins, Andrew Davidson, Judith Diers, Ruth Fischbach, Rachael Pine, Saumya RamaRao, June Reidenberg and Katie Schenk. The IRB meets five times each year. Each time a study is reviewed, the IRB determines whether an annual or more frequent review is appropriate. For more information, please visit http://popcouncil.org/what/ethics.asp.

## Competing interests

The authors declare that they have no competing interests.

## Authors' contributions

MMR participated in the study design. UR and BB were involved in all aspects of the study. All investigators contributed to the protocol and have read and approved its final version.

## Pre-publication history

The pre-publication history for this paper can be accessed here:

http://www.biomedcentral.com/1471-2458/11/257/prepub
